# Enhanced Fe-TiO_2_ Solar Photocatalysts on Porous Platforms for Water Purification

**DOI:** 10.3390/nano12061005

**Published:** 2022-03-18

**Authors:** Maria Leonor Matias, Ana Pimentel, Ana S. Reis-Machado, Joana Rodrigues, Jonas Deuermeier, Elvira Fortunato, Rodrigo Martins, Daniela Nunes

**Affiliations:** 1 CENIMAT|i3N, Department of Materials Science, School of Science and Technology, NOVA University Lisbon and CEMOP/UNINOVA, 2829-516 Caparica, Portugal; ml.matias@campus.fct.unl.pt (M.L.M.); acgp@campus.fct.unl.pt (A.P.); j.deuermeier@fct.unl.pt (J.D.); emf@fct.unl.pt (E.F.); 2 LAQV-REQUIMTE, Department of Chemistry, NOVA School of Science and Technology, Universidade NOVA de Lisboa, Campus de Caparica, 2829-516 Caparica, Portugal; ams.machado@fct.unl.pt; 3 Physics Department & I3N, Aveiro University, Campus Universitário de Santiago, 3810-193 Aveiro, Portugal; joana.catarina@ua.pt

**Keywords:** TiO_2_, photocatalysis, water purification, microwave synthesis, iron doping, porous platforms

## Abstract

In this study, polyethylene glycol-modified titanium dioxide (PEG-modified TiO_2_) nanopowders were prepared using a fast solvothermal method under microwave irradiation, and without any further calcination processes. These nanopowders were further impregnated on porous polymeric platforms by drop-casting. The effect of adding iron with different molar ratios (1, 2, and 5%) of iron precursor was investigated. The characterization of the produced materials was carried out by scanning electron microscopy (SEM), energy-dispersive X-ray spectroscopy (EDS), X-ray diffraction (XRD), X-ray photoelectron spectroscopy (XPS), and Raman spectroscopy. Optical characterization of all the materials was also carried out. SEM showed that pure TiO_2_ and Fe-TiO_2_ nanostructures presented similar nanosized and spherical particles, which uniformly covered the substrates. From XRD, pure TiO_2_ anatase was obtained for all nanopowders produced, which was further confirmed by Raman spectroscopy on the impregnated substrates. XPS and UV–VIS absorption spectroscopy emission spectra revealed that the presence of Fe ions on the Fe-TiO_2_ nanostructures led to the introduction of new intermediate energy levels, as well as defects that contributed to an enhancement in the photocatalytic performance. The photocatalytic results under solar radiation demonstrated increased photocatalytic activity in the presence of the 5% Fe-TiO_2_ nanostructures (Rhodamine B degradation of 85% after 3.5 h, compared to 74% with pure TiO_2_ for the same exposure time). The photodegradation rate of RhB dye with the Fe-TiO_2_ substrate was 1.5-times faster than pure TiO_2_. Reusability tests were also performed. The approach developed in this work originated novel functionalized photocatalytic platforms, which were revealed to be promising for the removal of organic dyes from wastewater.

## 1. Introduction

As a by-product of the society’s rapid development, environmental contaminants, such as dyes, drugs, and pesticides, are widely being released into effluents and represent, not only a risk to human health, but also to the environment, due to their complex structure and high recalcitrance. Spillage of dyes, widely used in various industries, such as water-soluble Rhodamine B (RhB) and Methylene blue (MB), endangers animals, plants, and human beings [1,2,3]. In this regard, a growing interest in the removal of pollution effluents from wastewater through environmentally friendly routes has spurred intensive research over the last few years [4,5,6].

Among the various techniques for decomposition and detoxification of organic dye effluents, photocatalysis is considered an appealing and inexpensive technology, which has been successfully employed for the treatment of pollutants and remediation of water. One of its advantages is the use of solar energy, which makes the process economically viable for large scale applications, while acquiring additional environmental value [2,3,7,8,9].

Concerning photocatalysts, TiO_2_ is in the spotlight and the subject of intense research, due to its remarkable properties, which include a strong oxidation potential to decompose organic pollutants, physical and chemical stabilities, low cost, non-toxicity, and earth abundance [10,11,12,13,14,15]. TiO_2_ is widely used in photocatalysis for the degradation of contaminants, in both aqueous and gaseous mediums [16].

TiO_2_ is a semiconductor that presents a wide band gap (~3.2 eV for anatase, ~3.0 eV for rutile and 3.4 eV for brookite [17], at room temperature) and is usually excited with high-energy UV photons, above the semiconductor’s band gap [18,19]. As a result, pure TiO_2_ absorbs in the UV region (4–5% of the solar spectrum [18]), which makes its photocatalytic efficiency low under visible light (visible region comprehends ~45% of the solar spectrum [20]). To overcome this issue and enhance the photocatalytic activity of TiO_2_-based materials, several approaches have been tested to extend their use under sunlight, including doping with metal and non-metal elements, through surface modification and by coupling with other semiconductor materials [12,16,20,21,22,23].

Doping TiO_2_ with transition metal ions has been reported to reduce the rate of electron-hole recombination, due to the formation of a Schottky barrier with TiO_2_. Simultaneously, it is responsible for narrowing the TiO_2_ band gap energy; thus, extending the absorption to the visible spectral range (red-shift), and leading to an improvement in the photocatalytic activity [24,25,26]. Doping could also potentially increase TiO_2_ crystallinity and enlarge the surface area [27]. Several metal ions have been successfully incorporated into a TiO_2_ lattice, including nickel [28], copper [29,30], chromium [31], iron [1], vanadium [32], and zinc [33].

Amongst the different metal ions, Fe appears an excellent candidate for doping TiO_2_, due to its half-filled d-electronic configuration [34,35] and the similar atomic radius of Fe^3+^ ion (0.69 Å) and Ti^4+^ ion (0.75 Å). The titanium positions in the TiO_2_ lattice can be easily substituted by the cation. Fe^3+^ is reported to provide trap centers for photo-generated electrons and holes, since the energy level of Fe^2+^/Fe^3+^ is located near Ti^3+^/Ti^4+^; thus, enhancing the charge separation [1,36,37]. Several studies reported the enhanced photocatalytic performance of Fe-doped TiO_2_ compared to other metals. For instance, Crisan et al., studied the effect of three transition metal ion dopants: Fe, Co, and Ni on TiO_2_ for the degradation of nitrobenzene on water. The best photocatalytic activity results were achieved with Fe-doped TiO_2_ nanopowders [38]. Ghasemi et al., also reported a comparison on the photocatalytic activity of pure and Cr, Mn, Fe, Co, Ni, Cu, and Zn-doped TiO_2_ for the degradation of Acid Blue 92 dye. Again, the most active photocatalyst was Fe-doped TiO_2_ [39,40]. Another study by Choi et al. [40,41] showed that among Fe^3+^, Mo^5+^, Ru^3+^, Os^3+^, Re^5+^, V^4+^,Rh^3+^, Co^3+^, and A1^3+^ metal ions, Fe^3+^ at 0.5 at.% exhibited the highest photocatalytic degradation of chloroform.

Nevertheless, in terms of the increase of the TiO_2_ photocatalytic activity with iron doping, some studies reported the opposite behavior. Multiple factors can contribute to the inconsistencies reported in the literature, including disparities in synthesis methods, annealing temperatures, the organic compounds used for the photocatalytic tests, and the concentration of the iron dopant [42,43,44].

Several methods have been employed for the synthesis of doped TiO_2_ based materials, including sol-gel [24,26], electrospinning, anodization [45], and microwave methods [46,47]. Microwave irradiation is a highly appealing method, which has been employed in several studies for its simplicity, homogeneous and fast heating, cost-effectiveness, selective heating, and facility of reaction scale-up [13,48,49,50,51,52]. Although several studies on the synthesis of Fe-doped TiO_2_ are reported in the literature, most of them employed methods that involve a considerable amount of time and a calcination step, which typically requires the use of high temperatures [53,54,55,56,57]. Regarding the microwave irradiation method, few studies have focused on a fast Fe-TiO_2_ microwave synthesis without the need to calcinate the samples; the approach developed in this study.

Crystallinity, porosity, surface chemical properties, and surface area of the metal oxides play an important role in the photocatalytic activity and, thus, need to be considered. Larger surface areas generally provide more active sites for reactions to occur, pore channels favor mass diffusion, and high crystallinity can improve metal oxides’ stability. Nevertheless, the challenge relies on the balance between crystallinity and the porous structure, since high crystallinity leads to a rearrangement and migrations of atoms, followed by a disarrangement of the porous structure, which, in consequence, causes a decrease in the specific surface area [58].

In order to produce improved materials with high surface area, several studies have focused on the synthesis of controlled porous TiO_2_-based materials with the addition of surfactants for the enhancement of photocatalytic activity [59,60,61,62]. Feng et al., reported enhanced metal-ion-doped TiO_2_ photocatalysts under solar light with a large BET (Brunauer–Emmett–Teller) surface area, synthesized by microwave irradiation, through the use of the surfactant P123 [59]. Kubiak et al., also demonstrated superior photocatalytic degradation of phenol and etodolac, with the nanomaterials obtained by microwave irradiation using different surfactants (PEG (Polyethylene glycol), Triton-X and P123) compared to pristine TiO_2_ samples [62]. In another research study, Jang et al., also explored the effect of Polyvinylpyrrolidone (PVP), Triton-X, and P123 on the fabrication of mesoporous structured TiO_2_ nanoparticles by a microwave-assisted sol-gel method. A higher photocatalytic performance for MB degradation was exhibited compared to P25, attributed to the large surface area and porosity obtained with the addition of surfactants [63].

Different substrates have been used for growing Fe-doped TiO_2_ nanostructures, including glass substrates [64,65], polyamide fabric [66], and Si substrates [67], but to the best of our knowledge, this has never been reported for porous water filters. The impregnation of these nanostructures on commercial water filters avoids the limitations and costs associated with the recovery of nanometer-sized particles.

In this study, pure TiO_2_ and 1, 2, and 5 mol% Fe-TiO_2_ nanostructures were synthesized by a fast surfactant-assisted microwave method (1 h), without any further calcination of the nanostructures, and impregnated using a simple drop-casting technique on polymeric substrates, used as water filters. Enhanced visible light absorption of TiO_2_-based materials on flexible porous water filter substrates was, thus, produced. The surface modification of TiO_2_-based materials with PEG was investigated. The materials produced were systematically characterized by XRD, Raman spectroscopy, SEM coupled with EDS, and XPS. Optical characterization was also carried out for the nanostructures, via UV–VIS absorption and room temperature diffuse reflectance measurements, to determine the optical band gap values. Finally, their efficiency as photocatalysts on water filters was investigated for the degradation of the organic model pollutant, RhB, under solar radiation.

## 2. Experimental Procedure

### 2.1. Preparation of Pure and Fe-TiO_2_ Nanostructures via Surfactant-Assisted Microwave Irradiation

Titanium (IV) isopropoxide (TTIP), 97% purity, was purchased from Sigma-Aldrich, St. Louis, MO, USA (CAS: 546-68-9) and was used as the titanium dioxide precursor. TTIP was dissolved in absolute anhydrous ethanol from Carlo Erba reagents, Val-de-Reuil, France (CAS: 64-17-5) and oxalic acid anhydrous from Sigma-Aldrich (CAS: 144-62-7). To prepare a solution of 120 mL, 5 mL of a 1 M oxalic acid solution was added to 115 mL of absolute ethanol and stirred for 10 min. Afterwards, 1 mL of polyethylene glycol (PEG 400) from Sigma-Aldrich (CAS: 25322-68-3) was added, followed by dropwise addition of 4 mL TTIP. Then the solution was stirred for 1 h. Microwave synthesis was performed using a CEM microwave digestion system, Matthews, NC, USA (MARS one), which was carried out at 1000 W, 170 ± 10 °C for 1 h. Solution volumes of 40 mL were transferred into Teflon vessels of 75 mL, which were kept sealed. To prepare Fe-TiO_2_ nanoparticles, 1, 2, and 5 mol% of iron (III) nitrate nonahydrate purchased from Sigma-Aldrich (CAS: 7782 81-8, 98% purity) were added to the previous solution, before the addition of TTIP. The nominal ratios of Fe precursor, TTIP, were 0.02, 0.03, and 0.07 for 1, 2, and 5 mol% of iron, respectively. The solution was stirred for 1 h, before transferring it to the microwave vessel. The as-synthesized TiO_2_ nanopowders were cleaned using a centrifuge at 4000 rpm for 5 min. The powders were washed repeatedly several times with deionized water, followed by isopropyl alcohol (IPA). Finally, the nanomaterials were kept overnight in a desiccator for drying, at 80 °C in vacuum.

### 2.2. Impregnation of Nanoparticles in Polymeric Substrates by Drop-Casting

**Figure 1:**
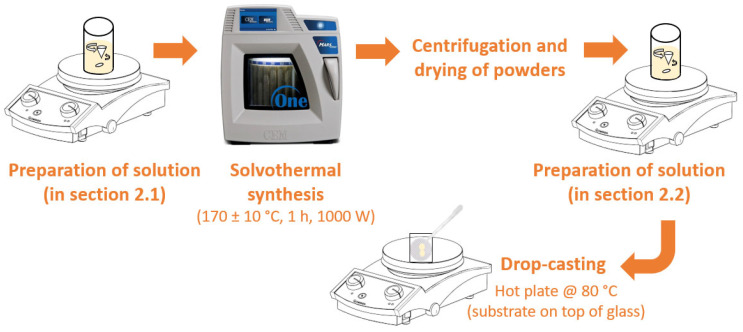
Schematic illustration for the synthesis of nanostructures and impregnation process.

In this study a simple and low-cost impregnation method was used, which had already been proved to be efficient for the functionalization of the substrates to be tested in photocatalytic experiments [68,69]. Several circular polymeric filters made of MCE (mixed cellulose esters, composed of inert cellulose nitrate and cellulose acetate polymers) with a pore size of 0.22 µm and diameter of 47 mm were tested. The filters were attached to previously cut ordinary glass squares using Kapton tape. These glass squares were used to prevent the polymeric substrates from bending, after complete drying of the droplets. The coffee-ring stain phenomenon of drop-casting can be suppressed by controlling the liquid properties, such as viscosity and pH, and by changing the temperatures of substrate and droplets [70,71,72]. In this study, 40 mg of nanoparticles was added to 9 mL of deionized water. Then, 1 mL of ethylene glycol from Carlo Erba Reagents (CAS: 107-21-1, 99.5% purity) was also added to the previous solution. It is reported that a small amount of ethylene glycol (10–30 vol.%) in colloidal suspensions produces uniform layers [73,74]. Before applying the droplets, and to mitigate the coffee-ring effect, the hydrophilic polymeric substrates were heated in a hot plate at 80 °C. Six layers of 1 mL of the aqueous solution were uniformly applied onto the substrates, leaving the necessary time for each layer to dry. A schematic illustration for the synthesis of the nanoparticles and the impregnation process is presented in Figure 1.

### 2.3. Photocatalytic Activity

The photocatalytic activities of pure TiO_2_ and Fe-TiO_2_ nanostructures on water filters were evaluated at RT, considering the degradation of RhB (C_28_H_31_ClN_2_O_3_) from Sigma-Aldrich under a solar light simulating source. The nanoparticles were impregnated on a substrate to aid in the recovery of nanosized materials. All the experiments were performed according to the international standard, ISO 10678 [49]. For each experiment, the polymeric-based materials were placed on the bottom of the reaction recipient and for each experiment, 50 mL of the RhB solution (5 mg/L) was stirred for 30 min in the dark, to establish absorption–desorption equilibrium. Solar light exposure was carried out by using a LED solar simulator LSH 7320, Irvine, CA, USA (AM 1.5 spectrum) with an intensity of 100 mW/cm^2^. The experiments were conducted under low constant magnetic agitation at 205 rpm. Absorption spectra were recorded using a PerkinElmer lambda 950 UV/VIS/NIR spectrophotometer, Waltham, MA, USA, with different time intervals, up to a total of 5 h. The measurements were performed in the 400–700 nm range every 30 min. The pristine substrate was also exposed with identical conditions.

The reusability experiments consisted in recovering the water filters with further discarding of the total liquid. The recovered filters were dried in air, prior to the next exposure. The recovered materials were then poured into fresh solution and exposed to solar light considering the same exposure times.

## 3. Characterization Techniques

X-ray Diffraction (XRD) measurements were carried out in a X’Pert PRO PANalytical powder (X’Pert diffractometer (Almelo, The Netherlands) using Cu Kα line radiation (λ = 1.540598 Å), operated at 45 kV and 40 mA). Diffraction patterns were recorded from 20° to 80° (detector angle 2θ) with a step of 0.05° in a Bragg–Brentano configuration, using the nanostructures in the form of powder. The simulated brookite corresponds to ICDD file No. 00-029-1360, simulated rutile to ICSD file No. 00-021-1276, and the simulated anatase to ICSD file No. 00-21-1272 with a = b = 3.7852 Å and c = 9.5139 Å. The average crystalline size of the synthesized nanomaterials was estimated using the Debye–Scherrer equation [75] with the software X’pert highscore plus (Almelo, The Netherlands, version 4.6a (4.6.1.23823)) [76].

Scanning electron microscopy (SEM) images were obtained using a Regulus 8220 Scanning Electron Microscope (Mito, Japan), while for the EDS analyses, a Carl Zeiss AURIGA CrossBeam FIB-SEM workstation (Oberkochen, Germany) was used. The dimensions of the nanoparticles were determined by measuring 60 nanostructures from SEM micrographs with ImageJ software (version 1.51j8).

X-ray photoelectron spectroscopy (XPS) measurements were carried out with a Kratos Axis Supra (Manchester, UK), using monochromated Al Kα irradiation (1486.6 eV). The acquisition of detailed scans was performed with an X-ray power of 225 W and a pass energy of 40 eV. All spectra were shifted to O 1 s at 530 eV [77]. Data analysis was carried out with CasaXPS.

Room temperature (RT) diffuse reflectance measurements, to obtain the optical bandgap, were performed using a Perkin Elmer lambda 950 UV/VIS/NIR spectrophotometer with a diffuse reflectance module (150-mm diameter integrating sphere, internally coated with Spectralon) and a powder sample holder. The calibration of the system was achieved using a standard reflector sample (reflectance, R = 1.00 from Spectralon disk). The reflectance (R) was obtained from 250 to 700 nm.

The absorption measurements were also carried out using the same UV/VIS/NIR spectrophotometer, and the measurements were performed in the 250–700 nm range.

Raman spectroscopy measurements were obtained with an inVia Qontor confocal Raman microscope from Renishaw (Kingswood, UK). A 17-mW He-Ne laser operated at 532 nm with a 10 s exposure time and settings of 5 accumulations. The Raman spectra were recorded in the range of 100–700 cm^−1^. The results presented for the impregnated substrates are based on the average of several points taken on their surface. All measurements were performed at room temperature.

## 4. Results and Discussion

Pure TiO_2_ and 1, 2, and 5 mol% of Fe-TiO_2_ nanostructures with PEG were successfully synthesized under microwave irradiation, considering a fast synthesis time (1 h). The characterization of the nanoparticles, in terms of morphology, crystalline structure, and optical properties was carried out. The as-synthesized nanopowders were further impregnated on water filters for photocatalytic tests. The addition of iron on TiO_2_, as well as the efficiency of the impregnation method was systematically investigated. Reusability tests within three repeated cycles were also performed.

### 4.1. Characterization of the Synthesized Nanoparticles

#### 4.1.1. SEM Analysis

**Figure 2:**
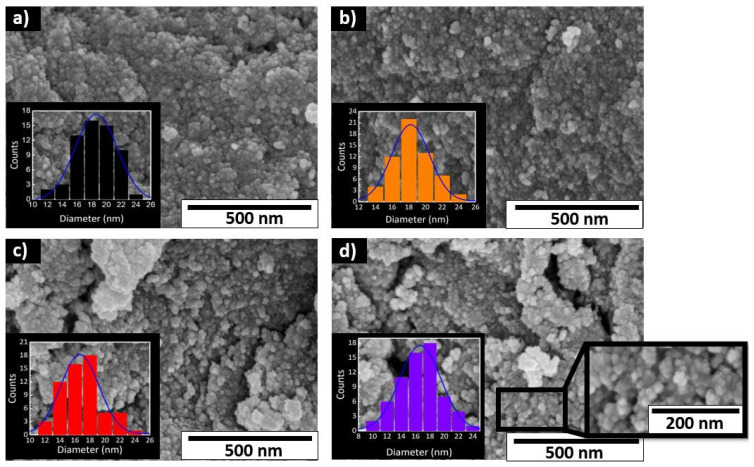
SEM images and insets showing the Gaussian model (in blue) for fitting the histograms of (**a**) pure TiO_2_, (**b**) 1 mol% of Fe-TiO_2_, (**c**) 2 mol% of Fe-TiO_2_, and (**d**) 5 mol% of Fe-TiO_2_ nanoparticles prepared by microwave irradiation using PEG.

Figure 2 depicts the SEM morphology of the pure TiO_2_ (Figure 2a) and 1, 2, and 5 mol% Fe-TiO_2_ nanoparticles (Figure 2b–d, respectively). PEG was used as a surfactant for all microwave syntheses. It can be observed that all conditions resulted in nanosized TiO_2_ particles with a spherical shape. These nanoparticles appear as agglomerates, since after drying the powder, there is aggregation in larger particles, reaching the micrometer range. The insets in Figure 2 also show the grain size histograms and the Gaussian model with an acceptable fitting for all particle size distributions. The estimated average particle sizes were 18.58 ± 2.73 nm, 18.40 ± 2.26 nm, 17.00 ± 2.57 nm, and 16.54 ± 3.02 nm for pure TiO_2_, and 1, 2, and 5 mol% of Fe-TiO_2_, respectively. A decrease in diameter was observed with the increase of iron content, which has been previously reported [56,78,79]. A more pronounced decrease in the nanoparticle size was achieved when the Fe concentration was above 2 mol%.

#### 4.1.2. XRD Analysis

**Figure 3:**
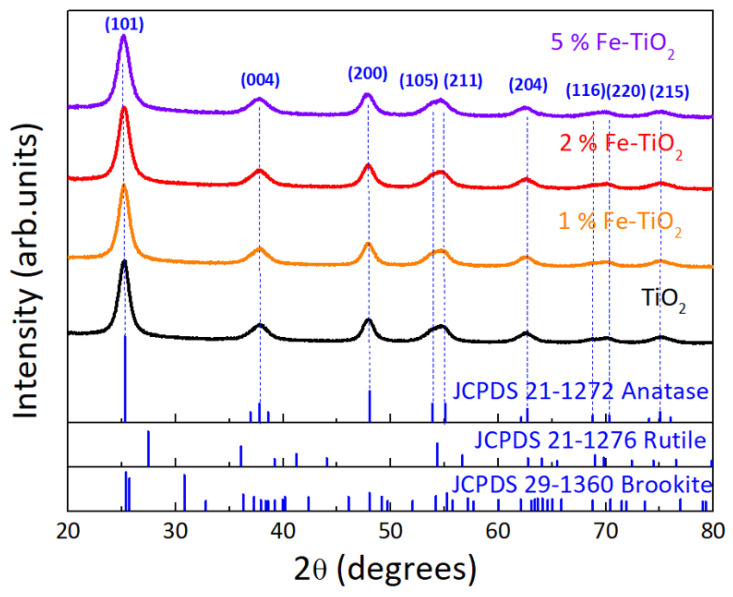
XRD diffractograms of TiO_2_ and 1, 2, and 5 mol% of Fe-TiO_2_ nanostructures with PEG synthesized by microwave irradiation. The simulated TiO_2_ anatase, rutile, and brookite structures are also presented for comparison.

The as-synthesized nanopowders were also investigated using XRD and the results are depicted in Figure 3. All peaks in the experimental diffractograms indicate the presence of TiO_2_ anatase phase, which has Ti^−6^ (octahedral) and O^−3^ (trigonal planer) coordination geometry [80,81]. The peaks detected correspond to the planes (101), (004), (200), (105), (211), (204), (116), (220), and (215) at 2θ = 25.3, 37.8, 48.0, 53.8, 54.9, 62.8, 68.9, and 75.0°, respectively. No peaks associated with rutile or brookite were detected. For the Fe-rich nanostructures, no peaks assignable to a metal oxide phase (Fe_2_O_3_ (hematite) or Fe_2_TiO_5_) were detected. As such, the quantity/percentage of these oxides could be too low to be detected, could be amorphous, or, since Fe^3+^ and Ti^4+^ have similar ionic radii, Fe^3+^ ions could have successfully substituted Ti^4+^ ions. No changes in peak intensity, as a function of iron loading, or shifts could be observed [1,49,82].

No peaks coming from impurities such as Ti(OH)_4_ were detected, and the XRD results demonstrate that the materials were well crystallized and highly nanostructured [49,52].

The average crystallite sizes of the synthesized nanomaterials were calculated using the Debye–Scherrer equation. The estimated crystallite sizes for each powder are summarized in Table 1.

As expected, the obtained values for the particle sizes obtained previously from SEM analysis (Section 4.1.1) are superior to the crystallite sizes, since particles are agglomerates of grains, and the grains are composed of several crystallites. Moreover, the increase of iron content led to a decrease in the crystallite and particle sizes, respectively. As reported, doping with Fe ions could inhibit the growth of TiO_2_ anatase crystals, leading to smaller particles [78,79,82]. Hence, it can be suggested that this effect is more pronounced for higher iron contents.

#### 4.1.3. XPS Analysis

The chemical composition of TiO_2_ and Fe-TiO_2_ nanostructures with PEG was analyzed by XPS. The conditions of 1 and 2 mol% of Fe-TiO_2_ showed a weak iron signal; therefore, these data were not included in this study; hence, only the composition of 5 mol% (corresponding to 1.4 at. %) is presented.

**Figure 4:**
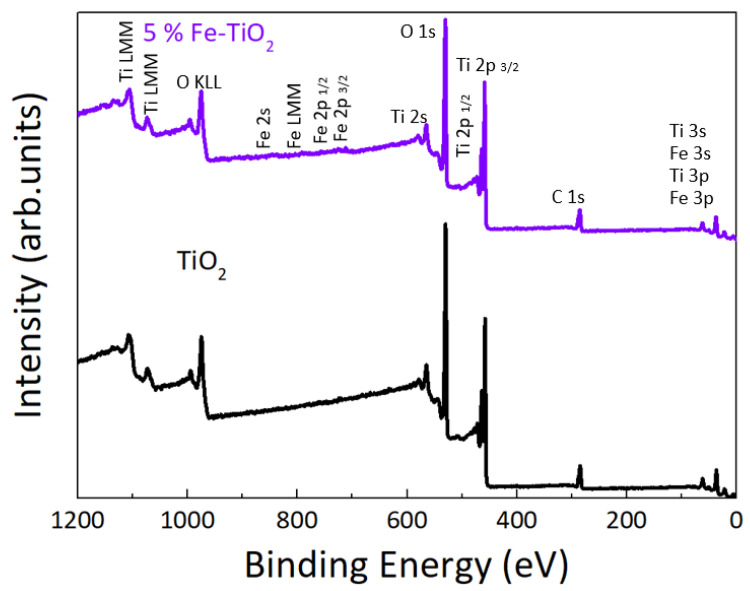
Survey spectra of pure TiO_2_ and Fe-TiO_2_ nanostructures with PEG.

**Figure 5:**
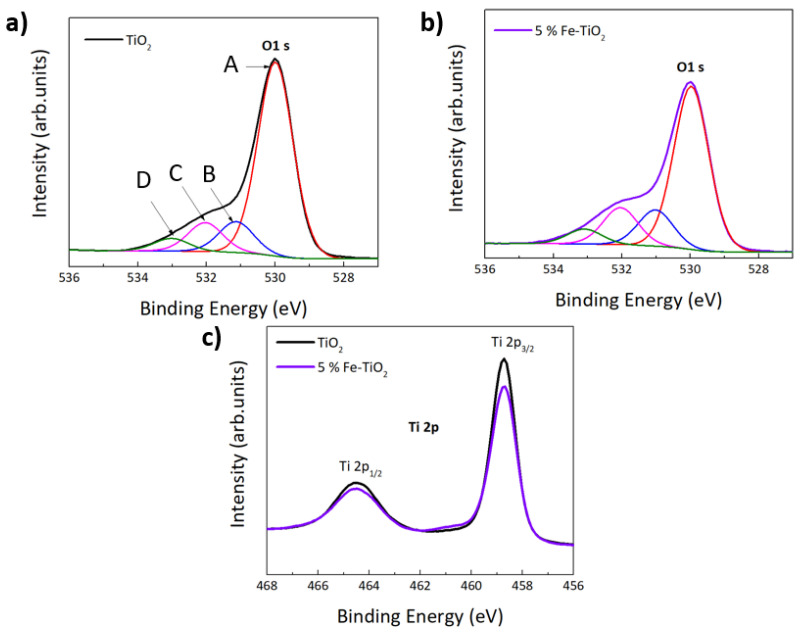
Deconvolution of XPS O 1s spectra of (**a**) pure TiO_2_, where features A, B, C and D are visible and correspond to lattice oxygen, undercoordinated oxygen either at the surface or close to oxygen vacancies and adsorbed water and organic species, respectively, and (**b**) 5% Fe-TiO_2_ nanostructures with PEG. Ti 2p peak is also shown in (**c**) for pure TiO_2_ and 5% Fe-TiO_2_ nanostructures with PEG.

Survey spectra of pure TiO_2_ and 5% Fe-TiO_2_ are shown in Figure 4. XPS results showed that Ti and O were clearly identified for pure TiO_2_, whereas for the Fe-TiO_2_ nanostructures, Fe could also be detected. A carbon peak was also visible, related to adventitious carbon at the surface. Figure 5 shows the high resolution XPS spectra of O 1s (deconvoluted) and Ti 2p core levels from pure synthesized TiO_2_ nanostructures with PEG and of 5% Fe-TiO_2_.

In Figure 5a,b, four peaks can be observed that correspond to lattice oxygen (A), undercoordinated oxygen either at the surface or close to oxygen vacancies (B), and adsorbed water and organic species (C,D) [83]. An increase of the peaks B–D with respect to peak A is observed in the Fe-TiO_2_, compared to TiO_2_. Part of this increase may be ascribed to a higher oxygen vacancy concentration, due to the doping. Due to charge neutrality, the substitution of titanium ions by iron of a lower oxidation state may lead to the formation of compensating defects, such as oxygen vacancies. In both cases, O 1s core level is centered at around 530 eV, which corresponds to the binding energy of the metal oxide (TiO_2_). A shoulder is observed at around 533 eV and is related to the OH groups at the surface [84]. Regarding Figure 5c, the two components of the Ti 2p core level that arise from spin orbit-splitting [85] can be observed for pure TiO_2_ and Fe-TiO_2_ nanostructures, both associated with the Ti^4+^ oxidation state. The peak positions and the peak separation of the Ti 2p doublet are in good agreement with previous studies of pure TiO_2_ nanostructures [86].

**Figure 6:**
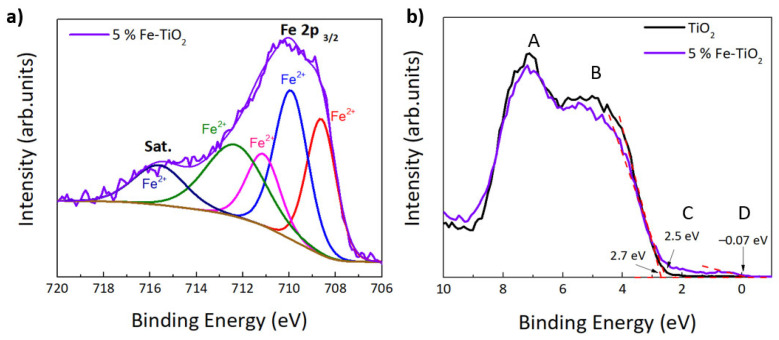
(**a**) XPS spectrum of Fe 2p for 5 mol% of Fe-TiO_2_ with PEG (in which red, blue, pink, green and dark blue colors represent the Gaussian–Lorentzian components that best fitted Fe 2p spectrum (GL 30)), and (**b**) valence band (VB) XPS spectra of pure TiO_2_ and Fe-TiO_2_ with PEG.

A detailed scan of Fe 2p from 5 mol % of Fe-TiO_2_, is shown in Figure 6a. The pronounced peak around 708.6 eV indicates the presence of Fe^2+^, and a set of fitting parameters accounting for the rich satellite structure of oxidized iron is available in the literature [87]. The deconvolution did not converge using the exact relative constraints for the peak parameter of Fe^2+^(FeO) in ref [87]. However, freeing the area of the peak at the highest binding energy led to convergence and a satisfactory fit quality. The intensity of this satellite peak at 5.9 eV from the center of gravity of the main peaks (another indication for Fe^2+^ [88]) is sensitive to the polarizability of the environment of the Fe atoms via the extra-atomic relaxation, one of the relaxation processes related to the photoemission event [89,90]. In contrast to FeO, Fe atoms in the Fe-TiO_2_ sample were surrounded by TiO_2_, which has about 40-times higher permittivity than FeO [91,92]. This justifies the different relative intensity of the satellite peak compared to FeO [87] and identifies the iron oxidation state as pure Fe^2+^. Photocatalytic reduction of Fe^3+^ to Fe^2+^ in a TiO_2_ matrix was speculated to occur in vacuum under X-ray irradiation [93], which made the quantification of the iron oxidation state in the original sample unreliable. Since the reduction of iron is the first part of the photocatalytic mechanism explained below, the detection of only Fe^2+^ by XPS corroborates the high photocatalytic activity of the sample.

In Figure 6b the XPS valence bands of pure TiO_2_ and Fe-TiO_2_ nanostructures are represented. No binding energy reference was applied to these spectra (C 1 s was measured at 285.8 eV). For Fe-TiO_2_ nanostructures, four features (A, B, C, and D) are visible at binding energy positions of ~7, 5, 2.5, and 1 eV, whereas pure TiO_2_ only shows features A and B. These values are consistent with the ones found in the literature [94]. Both A and B features are related to O 2p derived states and correspond respectively to the ‘bonding’ and ‘non-bonding orbital emissions’ of TiO_2_. Feature D is potentially related to the defect state of Ti^3+^ 3d. This would support the conclusion above, of a photocatalytic reduction in the XPS chamber [93]. At around 2.5 eV, another feature starts to appear with Fe doping (feature C), related to mixed Fe 3d and Ti 3d derived states very close to the Fermi level [84,94,95]. As seen in Figure 6b, pure TiO_2_ has a valence band maximum of ~2.5 eV, while for Fe-TiO_2_, a new state was formed within the band gap, with an edge of maximum energy at ~−0.07 eV. Additionally, Fe addition led to a shift towards lower binding energies for the maximum edge of the original valence band of TiO_2_ (from 2.7 eV for pure TiO_2_ to 2.5 eV for 5 mol% of Fe-TiO_2_) [96]. A similar shift was previously reported [94].

#### 4.1.4. Optical Characterization

**Figure 7:**
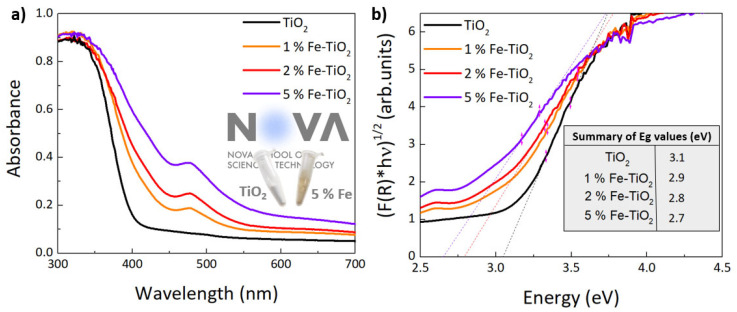
(**a**) Absorption curves and (**b**) optical band gaps estimation of TiO_2_ and 1, 2, and 5% mol of Fe-TiO_2_ catalysts with PEG synthesized by microwave irradiation.

To investigate the optical absorption characteristics of pure TiO_2_ and Fe-TiO_2_ nanostructures with PEG, UV-VIS absorption spectra were recorded (Figure 7a). Pure TiO_2_ shows an absorption peak at 354 nm (~3.5 eV) and with an onset around 400 nm (~3.1 eV), which is fairly in line with the anatase band gap excitation [97,98]. Moreover, undoped TiO_2_ also did not show any absorption beyond 400 nm. Meanwhile, for Fe-TiO_2_, even though the absorption maximum is placed at the same value obtained for pure TiO_2_ (which was also previously reported [99]), the onset of absorption is seen to shift towards higher wavelengths, due to the increasing contribution from a tail of states that extends from the absorption maximum. This observation is likely related to the higher density of defect states present in these materials, contributing to the visible light absorption. Such an increase accompanies the increase in the Fe%. This could, effectively, indicate that the visible light absorption of TiO_2_ has been enhanced by the introduction of Fe. Moreover, a small band can be distinguished at around 476 nm, becoming more pronounced with higher concentrations of iron. According to the literature, this visible light absorption can be attributed to two factors: One is the formation of a dopant energy level (Fe^3+^/Fe^4+^) within the band gap of TiO_2_, which is related to the excitation of 3d electrons of Fe^3+^ from the dopant energy level to the TiO_2_ conduction band at 415 nm. The second can be ascribed to the d–d transition of Fe(III) or the charge transfer transition between interacting iron ions (broad band at around 500 nm) [44,100]. Considering the XPS result discussed above, the introduction of new intermediate levels at the nanoparticles’ surface may be a likely explanation for the emergence of this new absorption band. These levels can act as traps and reduce the rate of recombination [82,101]. Besides that, a further reduction of the nanoparticles’ size could accentuate their contribution from surface defects, due to the increase in the surface/volume ratio. The enhancement of visible light absorption can also be seen from the inset of Figure 7a. The powder color changed from white (pure TiO_2_ on the left side) to yellow (Fe-TiO_2_ on the right side). All of these reasons may, thus, contribute to an improvement in the photocatalytic efficiency of the nanomaterials under visible light.

The optical band gap energy ($E_{g}$) values were estimated based on the UV-VIS diffused reflectance and can be expressed by a Tauc equation and Kubelka–Munk function, represented in Equations (1) and (2):

(1) $${\left(F\left(R\right)hv\right)}^{m}=A\left(hv-E_{g}\right)$$

(2) $$F\left(R\right)=\frac{\left(1-R^{2}\right)}{2R}\;\frac{K}{S}$$

where R is the reflectance of an infinitely thick specimen, K and S are the absorption and scattering coefficients, respectively [102], hv is the photon energy, A is a proportionality constant, and m is a constant exponent, which determines the type of optical transitions (m = 2 is for direct allowed transitions, and m = 1/2 for indirect transitions). The band gap values can be obtained by extrapolating the linear part of the plots relating ${\left(F\left(R\right)hv\right)}^{m}$ and hv to ${\left(F\left(R\right)hv\right)}^{m}$ = 0 [56,102]. Anatase TiO_2_ has an indirect band gap, and so m = 1/2. Figure 7b shows a band gap reduction with an increase of iron concentration, when compared to the pure TiO_2_ band gap. The obtained band gap values were 3.1 eV for pure TiO_2_, decreased to 2.9 eV for 1 mol% Fe-TiO_2_, followed by 2.8 and 2.7 eV for the 2 and 5 mol% Fe-TiO_2_ nanopowders, respectively. An acceptable agreement was found with earlier studies, in which TiO_2_ anatase was reported to have an optical band gap of around 3.2 eV, while for the TiO_2_ doped with iron, the value gradually decreased when increasing the dopant concentration [103]. This reduction is probably associated with the formation of new intermediate energy levels [82].

### 4.2. Characterization of the Impregnated Substrates

**Figure 8:**
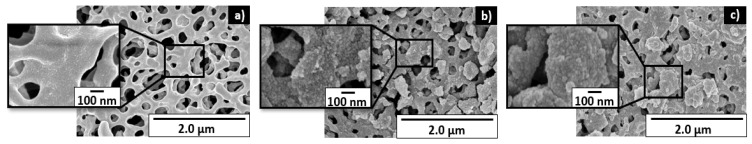
SEM images of the pristine porous polymeric substrates, together with the impregnated ones. (**a**) Pristine substrate (without nanopowders), (**b**) impregnated with pure TiO_2_ and with (**c**) 5 mol% of Fe-TiO_2_ nanopowders with the addition of PEG.

As previously mentioned, the as-synthesized nanopowders were impregnated on porous water filters, to aid in the recovery of the nanosized photocatalysts. Pure TiO_2_ and 5 mol% of Fe-TiO_2_ nanopowders were incorporated on the substrates for further photocatalytic experiments. Figure 8 shows the SEM images of the pristine porous substrates, together with the impregnated ones. From Figure 8, it is possible to compare the differences between the substrates, without (Figure 8a) and with the nanopowders (Figure 8b,c). The substrate presents a micro-sized porosity, which remained after impregnation. Regarding the impregnated substrates, the nanopowders of pure TiO_2_ (Figure 8b) and 5 mol% of Fe-TiO_2_ (Figure 8c) formed, by using the drop-casting technique, uniform films of nanoparticles that entirely and uniformly covered the substrates. Some micrometer-sized agglomerates were also observed on the substrate’s surfaces. In terms of morphology, and as expected from the SEM analysis of nanopowders, similar nanosized and spherical particles were obtained on the investigated porous substrates in both conditions.

EDS analyses were also carried out on these substrates, as shown in Figure S1. Figure S1a shows the pristine substrates that were only composed of C and O, consistent with a polymeric substrate, Figure S1b,c, respectively. For the impregnated substrates with pure TiO_2_ nanoparticles (Figure S1d), a strong presence of Ti (Figure S1g) is observed, together with C and O (Figure S1e,f). In the case of impregnated substrates with 5 mol% of Fe-TiO_2_ nanoparticles (Figure S1h), the presence of Ti (Figure S1k) along with Fe (Figure S1l)) is visible, as well as C (Figure S1i) and O (Figure S1j), which confirms the successful impregnation of the substrates. In EDS analyses, the presence of some micrometer-sized agglomerates on the impregnated substrates was visible, as well as the homogeneous distribution of all elements. 

The EDS chemical analysis of the 5 mol % Fe-TiO_2_ nanopowders impregnated on the porous substrate is summarized in Table 2.

The EDS Fe:Ti atomic ratio obtained was 0.05. This value is in line with the ratio obtained by XPS (0.06).

**Figure 9:**
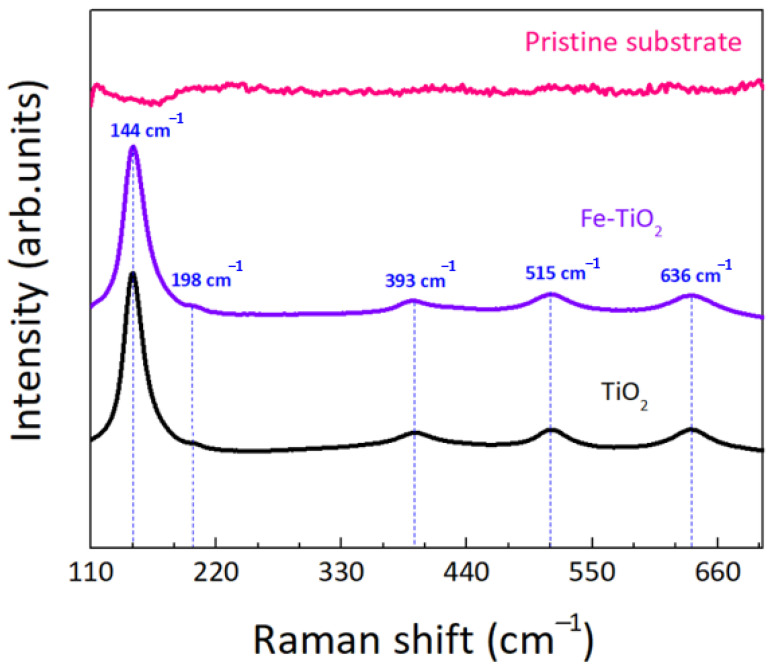
Raman spectra of the pristine polymeric substrate, TiO_2_ and Fe-TiO_2_ nanopowders impregnated on the polymeric substrates. Vertical dotted lines represent anatase TiO_2_ bands.

Raman measurements were also carried out, as this technique allows distinguishing between TiO_2_ phases [104] and evaluating the purity of TiO_2_ and Fe-TiO_2_ nanostructures impregnated on polymeric substrates. The obtained results are presented in Figure 9.

The Raman spectrum of the pristine substrate is also shown for comparison, demonstrating that there was no contribution associated to the substrate. In the impregnated substrates, five bands corresponding to six active modes of the tetragonal anatase phase can be observed in Figure 9, i.e., 144 cm^−1^ (E_g_), 198 cm^−1^ (E_g_), 393 cm^−1^ (B_1g_), 515 cm^−1^ (B_1g_ + A_1g_), and 636 cm^−1^ (E_g_) [52,105,106]. The Eg mode is ascribed to symmetric stretching vibration in octahedral TiO_6_ clusters, while the B_1g_ mode is related to symmetric bending vibration in the same clusters. The A_1g_ mode is assigned to anti-symmetric bending vibration and it is reported that the E_g_ mode at 636 cm^−1^ is attributed to the displacement of symmetric oxygen atoms in Ti-O bonds in the x,y-plane [107,108]. No additional peaks related to iron or iron oxides were detected in the Fe-TiO_2_ impregnated substrate, which corroborates the results of XRD.

### 4.3. Photocatalytic Performance

**Figure 10:**
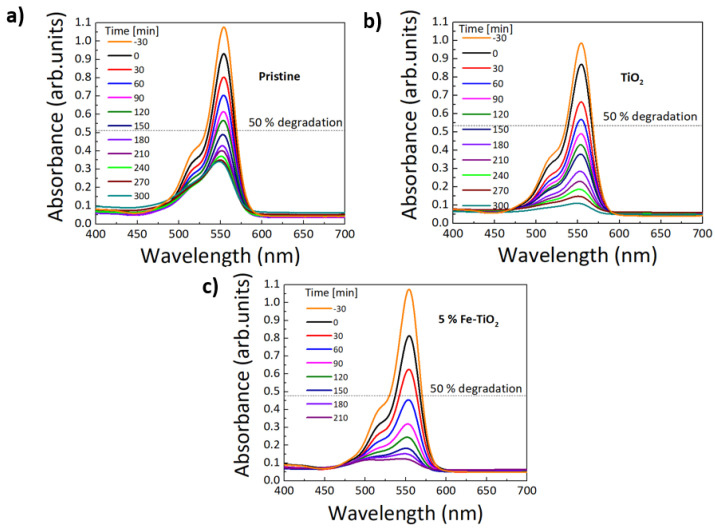
RhB absorbance spectra under simulated solar light radiation (LED simulator with AM 1.5 spectrum) up to 5 h for the polymeric substrates (**a**) without nanopowders, (**b**) with pure TiO_2_, and (**c**) with 5 mol% of Fe-TiO_2_ nanopowders with the addition of PEG.

Absorbance spectra were acquired to evaluate the photocatalytic activity of the impregnated substrates in the degradation of RhB under solar radiation at room temperature (Figure 10). The absorption peak intensity of RhB (occurring at 554 nm [109]) was measured at different irradiation times, to estimate the decrease of RhB content. The photodegradation rate of the RhB can be calculated using the following Equation (3):

(3) $$\mathrm{Degradation}\;\left(\%\right)=\frac{A_{0}-A}{A_{0}}\times 100$$

where $A_{0}$ is the light absorbance of the RhB solution before irradiation and after absorption–desorption equilibrium in the dark, and A is the light absorbance of the RhB solution after irradiation [110,111].

According to Figure 10a, without a catalyst on the surface of the polymeric substrates, some degradation of the dye was observed, both in the absence and presence of light. As reported in the product description, these polymeric membranes can also be used in aqueous solutions as filters. They possess a high porosity percentage and, thus, act like hydrophilic ‘sponges’ that can easily capture the RhB dye molecules on the pore sites in a few hours (63% of degradation was achieved in 5 h). This retention of RhB dye molecules is also clearly observed in Figure S2, with the color change of the substrate before (from white, Figure S2a,b) and after photocatalysis (to fuchsia pink, Figure S2c). Nevertheless, since all experiments were conducted with the same substrate, this contribution will be equal for all the materials studied [52]. In the presence of pure TiO_2_ (Figure 10b), a higher degradation percentage of 87% was achieved for the same time exposure as the substrate without catalyst. On the other hand, the addition of 5 mol% of Fe drastically improved the photocatalytic activity of TiO_2_. In 3.5 h, a RhB degradation of 85% was obtained, whereas for pure TiO_2_, the RhB degradation was 74% for the same exposure time.

As mentioned before, various factors can influence photocatalytic activity, such as the crystalline structure, morphology, porosity, particle size, iron content, and the presence of surface defects [53,112]. Regarding the contribution of the particles’ shape and size to the enhanced photocatalytic behavior observed, and as assessed by the SEM images in Figure 2 and estimated from the XRD results, all conditions resulted in spherical nanoparticles in the same size range (around ~18 nm from SEM measurements). Since both the size and shape of the nanoparticles are comparable, similar contributions are expected for all materials; thus, this does not explain the observed behavior for the 5% Fe-TiO_2_ material.

Moreover, in terms of the TiO_2_ phase present, it is well known that anatase TiO_2_ shows superior photocatalytic activity to rutile or brookite [113,114]. It has been reported that anatase exhibits a longer lifetime of photogenerated electrons and holes than rutile and brookite [115]. Additionally, the average effective mass of electrons and holes in anatase is smaller than that of rutile and brookite, which favors a faster migration of photogenerated charge carriers from the interior to the surface of anatase; thus, reducing the recombination rate and improving the photocatalytic activity [115]. As revealed by the XRD and Raman spectroscopy results, all synthesized materials have a anatase TiO_2_ phase, hence the photocatalytic degradation enhancement of the substrate with Fe-TiO_2_ must be owing to the introduction of iron ions into the TiO_2_ lattice.

The enhanced photocatalytic behavior observed with the Fe addition may be ascribed to the mechanism of the photocatalytic processes in Fe-TiO_2_ material. It is reported that there are two energy levels: the reduction level, which is located below the TiO_2_ conduction band (Fe^3+^/Fe^2+^), and another one, the oxidation level, above the TiO_2_ valence band (Fe^3+^/Fe^4+^). First, the formation of Fe^2+^ species occurs due to the migration of photogenerated electrons from TiO_2_ to Fe^3+^. These Fe^2+^ species are unstable, owing to the loss of the d^5^ electronic configuration, and easily oxidize to Fe^3+^ by transferring electrons to absorbed O_2_ and forming reactive superoxide anions (O_2_^−^). Meanwhile, Fe^3+^ ions can act as a hole trap, since the Fe^3+^/Fe^4+^ energy level is above the TiO_2_ valence band and oxidize to Fe^4+^. Fe^4+^ then reduces to Fe^3+^ by reacting with an OH^−^ group, and hydroxyl radicals (•OH) are formed. These hydroxyl radicals are powerful oxidizing species, which attack the chemical bonds of the surface-adsorbed organic materials. Therefore, Fe^3+^ ions can act as trap sites for the photogenerated electrons and holes; thus, suppressing the recombination of those photogenerated charges and ultimately enhancing the photocatalytic activity under visible light [35,56].

However, an important note should be made regarding the amount of iron content that plays an important role in photocatalytic activity [103]. Some studies reported, for instance, a reduction in the photocatalytic activity when the dopant concentration increases. It is stated that multiple trappings of charge carriers take place, which in consequence increases the electron–hole recombination. Thus, fewer charge carriers will be able to reach the surface to degrade the pollutant. Moreover, an excessive concentration of dopant may accumulate on the surface of the catalyst, reducing the penetration depth of light and, consequently, the number of active sites [35]. Nevertheless, this excessive dopant trend does not seem to occur in this study, since improved photocatalytic activity was demonstrated relative to pure TiO_2_.

The generation of intermediate energy levels, as a result of the presence of Fe ions was also confirmed by XPS, which could have served as trapping centers for the photogenerated carriers. As revealed by XPS, the valence state of Fe ions is lower than that of lattice Ti ions. Considering charge balance, oxygen vacancies are likely to have existed in the Fe-TiO_2_ sample and are also expected to play important roles in enhanced photocatalysis, since they can act as photoinduced charge traps and adsorption sites; thus, contributing to increasing the lifetime of photogenerated charge carriers and resulting in an improvement of the photocatalytic performance [116,117,118].

**Figure 11:**
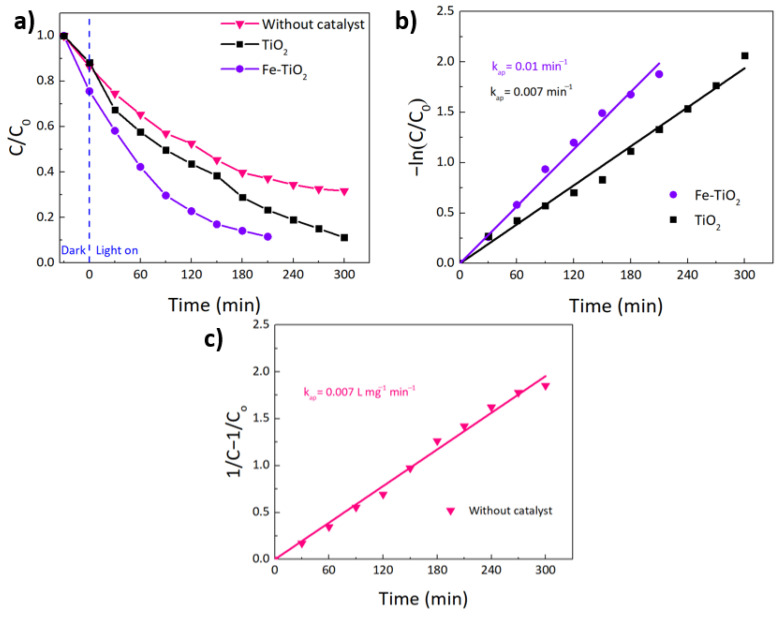
(**a**) RhB degradation ratio (C/C_0_) vs. exposure time for the impregnated substrates (with pure TiO_2_ and 5 mol% of Fe-TiO_2_ photocatalysts) and the pristine substrate (without catalyst), under a sunlight simulator. (**b**) Pseudo-first order kinetics for RhB photocatalytic degradation of the investigated impregnated substrates. (**c**) Pseudo-second order kinetics for RhB photocatalytic degradation of the pristine substrate (without catalyst).

Comparing the curves in Figure 11a, it can also be confirmed that the photodegradation was much faster with the Fe-TiO_2_ substrate. The reaction kinetics were also investigated using the Langmuir–Hinshelwood kinetic model. This model can be simplified to a first-order kinetics reaction (4):

(4) $$\ln\left(\frac{C}{C_{0}}\right)=-k_{\mathrm{ap}}t$$

where $k_{\mathrm{ap}}$ is the photodegradation apparent rate constant, t is the time, $C_{0}$ is the initial concentration, and C is the concentration at a certain time [111,112]. According to the Lambert–Beer law, the concentration is proportional to the absorbance; thus, it can be assumed that $\ln\left(\frac{C}{C_{0}}\right)\;\propto \;\ln\left(\frac{A}{A_{0}}\right)\;$ [119].

Based on (4), the rate constants $k_{\mathrm{ap}}$ (min^−1^) can be determined by plotting $-\ln\left(\frac{C}{C_{0}}\right)\;\mathrm{or}-\ln\left(\frac{A}{A_{0}}\right)\;$ versus time (Figure 11b) [119]. From the slope of the linear regressions, the rate constants can be obtained. The obtained kinetic parameters (rate constants, linear regression coefficients, and half-life times) are summarized in Table 3. From Figure 11b and Table 3, it can be concluded that the photocatalytic dye degradation follows the first order-kinetics for both TiO_2_ and Fe-TiO_2_ samples, as the correlation constant (R^2^) for the fitted lines is above 0.95 [111]. The obtained photodegradation apparent rate constants were found to be 0.007 min^−1^ and 0.01 min^−1^ for the substrates with pure TiO_2_ and 5% Fe-TiO_2_ nanostructures, respectively. The obtained *k*_ap_ values indicate that the RhB photodegradation with Fe-TiO_2_ materials was 1.5-times faster than pure TiO_2_.

In contrast, the pristine substrate did not reveal a good fit for the pseudo-first-order kinetics equation. In this case, the data showed a satisfactory fitting (R^2^ = 0.99), by using a pseudo-second order equation. Equation (5) indicates that the reaction rate is proportional to the concentration of the reactant:

(5) $$\frac{1}{C}-\frac{1}{C_{0}}=k_{\mathrm{ap}}t$$

Based on (5), the rate constant can be obtained by a linear fit, through the plot of $\frac{1}{C}-\frac{1}{C_{0}}$ versus time [120]. Figure 11c shows the fitting of the pristine substrate using a pseudo-second order equation. This kinetic reaction trend can be explained by the fast absorption intensity of RhB during the first 120 min, followed by a decrease and stabilization of absorption. Since kinetic rate coefficients are impossible to compare for different pseudo-order processes, half-life times (t_1/2_) were determined. Half-life times are defined by the time it takes for the concentration of a reactant to reach half of its initial value [121], and can be determined using Equations (6) and (7):

(6) $$t_{1/2}=\frac{2^{n_{a}-1}-1}{\left(n_{a}-1\right)k_{\mathrm{ap}}{\left[A_{0}\right]}^{n_{a}-1}}$$

For $n_{a}$ = 1:

(7) $$t_{1/2,}n_{a}\;{{\;}_{=}}_{1}=\frac{\ln\left(2\right)}{k_{\mathrm{ap}}}$$

where $n_{a}$ is the apparent or pseudo reaction order, $A_{t}$ is the ratio of the concentration at a given time (t) to initial concentration (C/C_0_), thus $A_{0}\;$= 1 [121,122].

As expected, the kinetic studies confirm that Fe-TiO_2_ impregnated substrate exhibited the highest photocatalytic degradation of RhB, when compared to pristine and TiO_2_-impregnated substrates under the same experimental conditions, since it achieved the lowest half-life time.

### 4.4. Reusability Tests

Reusability tests of the photocatalytic materials are of great importance, to check their applicability in real wastewater treatments. To evaluate the possibility of reuse, the best photocatalyst, i.e., 5 mol% of Fe-TiO_2_ photocatalyst on the porous substrate, was chosen for the experiments. Five consecutive cycles of RhB photocatalytic degradation were performed under the same experimental conditions and up to 210 min (3.5 h). On Figure S3, the reusability of the pristine substrate is also presented (up to 3 cycles).

**Figure 12:**
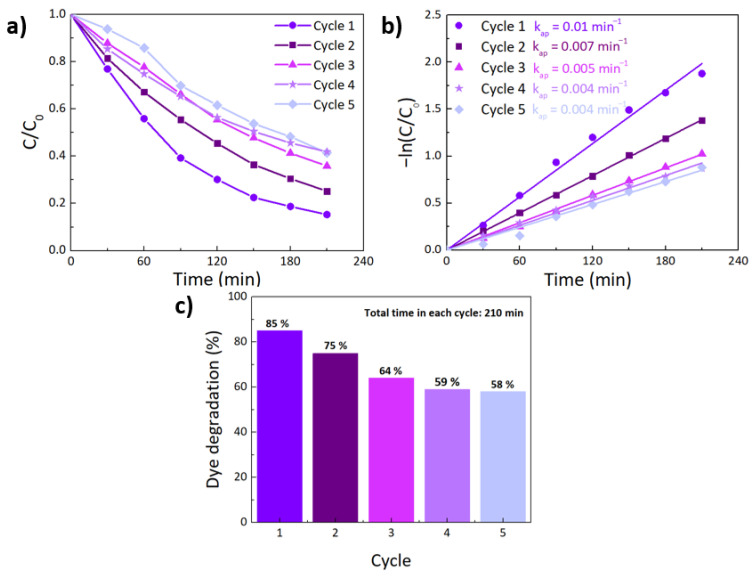
(**a**) Different cycles of RhB degradation ratio (C/C_0_) vs. exposure time for 5 mol% of Fe-TiO_2_ on the porous substrate, (**b**) pseudo-first-order kinetics for RhB photocatalytic degradation of the impregnated investigated substrate (with 5 mol% of Fe-TiO_2_), and (**c**) reusability of 5 mol% of Fe-TiO_2_ over three degradation cycles under solar simulator light.

In Figure 12a,b, a decrease in the photocatalytic activity is observed. The reaction rate decreased with the number of uses, from 0.01 min^−1^ in the first cycle, to 0.004 min^−1^ in the fifth cycle.

The reusability results can also be seen in Figure 12c. The impregnated substrate lost approximately 27% of its degradation efficiency after performing five repeated cycles, since in the first cycle a dye degradation of 85% was achieved, while in the fifth cycle it was 58%. Despite the considerable efficiency loss after three cycles (of around 21%), a much smaller efficiency loss was observed during the next cycles. This loss could be attributed to the high adsorption phenomenon of RhB, where RhB molecules remain adsorbed on the photocatalyst surface, hindering the available pore sites of the substrate with the nanostructures for reaction [123,124]. The reusability tests of the pristine substrate confirmed that a significant contribution to this loss of degradation efficiency comes from the substrate, with the adsorption of RhB molecules through the cycling tests (see Figure S3). This phenomenon also occurs on the impregnated substrates decreasing their overall cycling efficiency.

## 5. Conclusions

Pure TiO_2_ and Fe-TiO_2_ nanostructures were successfully synthesized by a fast surfactant-assisted microwave irradiation (1 h), without a calcination step, and impregnated by drop-casting on porous polymeric substrates. The approach used in this study enabled the total covering of the porous substrates and the evaluation of their photocatalytic activity in the degradation of RhB under solar radiation. SEM confirmed the formation of fine particles with a sphere-like appearance and films that uniformly coated the substrates. XRD revealed the presence of pure TiO_2_ anatase in all nanostructures, which was further confirmed by Raman spectroscopy on the impregnated substrates. The platforms impregnated with 5 mol% of Fe-TiO_2_ nanostructures exhibited an enhanced RhB photodegradation, when compared to pure TiO_2_ and the pristine substrates. The highest photocatalytic activity for the Fe-TiO_2_ material under solar radiation reached 85% after 3.5 h, compared to 74% with pure TiO_2_. The photodegradation rate of RhB dye with the Fe-TiO_2_ substrate was 1.5-times faster than pure TiO_2_. The XPS and UV-VIS results support that the presence of Fe ions led to the introduction of new energy levels, as well as defects, such as oxygen vacancies, that played an important role as traps for the photogenerated carriers, leading to a reduction in the recombination rate, followed by a visible enhancement in the photocatalytic activity. In summary, this study demonstrated that the synergy between the micro-porosity of the substrates and the surface-modified Fe-TiO_2_ nanostructures enhanced the substrates’ photocatalytic properties. Flexible and eco-friendly photocatalytic functionalized substrates were produced with potential for wastewater removal.

## Figures and Tables

**Table 1:** Estimated crystallite sizes (nm) for TiO_2_, 1, 2, and 5% mol of Fe-TiO_2_ nanoparticles with PEG synthesized by microwave irradiation.

	TiO_2_	1% Fe-TiO_2_	2% Fe-TiO_2_	5% Fe-TiO_2_
Sizes (nm)	6.0	5.7	5.2	4.9

**Table 2:** EDS chemical analysis of the 5 mol% Fe-TiO_2_ nanopowders after impregnation on the porous substrate.

Elements	At. %
C	32.8
Ti	26.8
O	39.0
Fe	1.40
Total	100

**Table 3:** Kinetic parameters (rate constants *k*_ap_, linear regression coefficients R^2^, and half-life times t_1/2_) for RhB photocatalytic degradation under solar radiation with the pristine substrate (without catalyst) and the impregnated materials (with pure TiO_2_ and 5 mol% of Fe-TiO_2_ photocatalysts).

		*k*_ap_ (min^−1^)	*k*_ap_ (L mg^−1^ min^−1^)	R^2^	Half-Life Time t_1/2_ (min)
Pseudo-second order reaction fitting	Pristine	-	0.007	0.99	143
Pseudo-first order reaction fitting	TiO_2_	0.007	-	0.99	99
	5% Fe-TiO_2_	0.01	-	0.98	69

## Data Availability

The authors confirm that the data supporting the findings of this study are available within the article and its Supplementary Materials.

## Supplementary Materials

The following supporting information can be downloaded at: https://www.mdpi.com/article/10.3390/nano12061005/s1, Figure S1: SEM images of the impregnated polymeric substrates (a) pristine (without nanopowders) (d) with pure TiO_2_ and (h) with 5% Fe-TiO_2_ nanopowders with the addition of PEG. The corresponding EDS maps of C (b,e,i), O (c,f,j), Ti (g,k) and Fe (l) are also visible; Figure S2: Porous polymeric substrates (water filters) used in this study (a) pristine (b) impregnated substrate before photocatalysis and (c) impregnated substrate after photocatalysis; Figure S3: Reusability of pristine substrate over 3 degradation cycles under solar simulator light.
