# Supplementary material for: Enhanced Fe-TiO2 Solar Photocatalysts on Porous Platforms for Water Purification
Source: Nanomaterials (Basel). 2022 Mar 18;12(6):1005. doi: 10.3390/nano12061005 (PMC8955547; doi:10.3390/nano12061005)
Supplement: Supplementary file 1 [file nanomaterials-12-01005-s001.zip › nanomaterials-1601940-supplementary.pdf]

Supplementary Materials

Enhanced Fe-TiO_2 Solar Photocatalysts on Porous Platforms for Water Purification

Maria Leonor Matias [1], Ana Pimentel [1], Ana S. Reis-Machado [2], Joana Rodrigues [3], Jonas Deuermeier [1], Elvira Fortunato [1], Rodrigo Martins [1,*] and Daniela Nunes [1,*]

[1] CENIMAT|i3N, Department of Materials Science, School of Science and Technology, NOVA University Lisbon and CEMOP/UNINOVA, Caparica, 2829-516, Portugal; ml.matias@campus.fct.unl.pt (M.L.M.); acgp@campus.fct.unl.pt (A.P.); j.deuermeier@fct.unl.pt (J.D.); emf@fct.unl.pt (E.F.)
[2] LAQV-REQUIMTE, Department of Chemistry, NOVA School of Science and Technology, Universidade NOVA de Lisboa, Campus de Caparica, 2829-516 Caparica, Portugal; ams.machado@fct.unl.pt
[3] Physics Department & I3N, Aveiro University, Campus Universitário de Santiago, 3810-193 Aveiro, Portugal; joana.catarina@ua.pt
* Correspondence: rm@uninova.pt (R.M.); daniela.gomes@fct.unl.pt (D.N.); Tel.: +351-212948562 (R.M. & D.N.); Fax: +351-21-294-8558 (R.M. & D.N.)

Figure S1 shows the EDS analysis of the pristine porous water filters together with the impregnated ones.

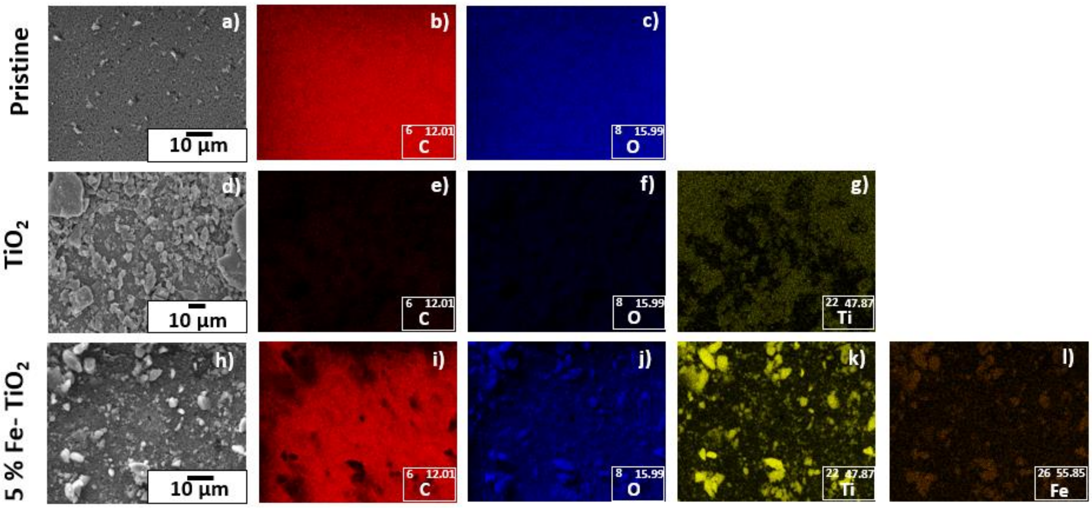

Figure S1. SEM images of the impregnated polymeric substrates (**a**) pristine (without nanopowders) (**d**) with pure TiO_2 and (**h**) with 5% Fe-TiO_2 nanopowders with the addition of PEG. The corresponding EDS maps of C (**b**,**e**,**i**), O (**c**,**f**,**j**), Ti (**g**,**k**) and Fe (**l**) are also visible.

Figure S2 shows the real image of the water filters used for experiments. It is evident that the impregnation does not change the general aspect of the substrate, however after photocatalysis, an evident change of color is observed. The shrink of the substrate is related to the drying process after the contact with liquid.

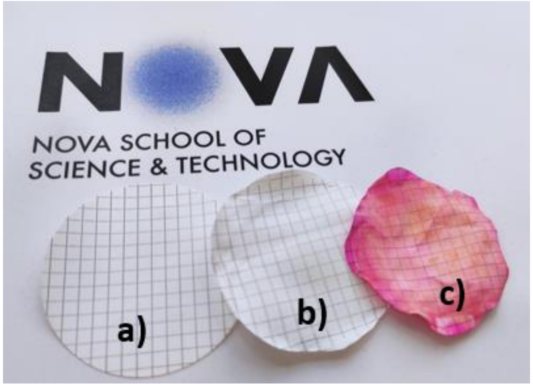

Figure S2. Porous polymeric substrates (water filters) used in this study (**a**) pristine (**b**) impregnated substrate before photocatalysis and (**c**) impregnated substrate after photocatalysis.

Figure S3 shows the reusability tests of the pristine substrate over 3 degradation cycles under solar simulator light.

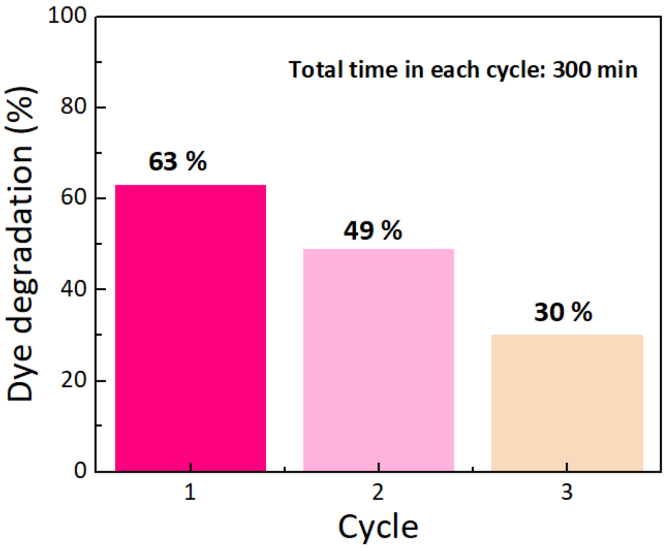

Figure S3. Reusability of pristine substrate over 3 degradation cycles under solar simulator light.
